# Pelvic floor hypertension: possible factors for pelvic floor tenderness in endometriosis patients—a pilot study

**DOI:** 10.1007/s00404-023-07192-5

**Published:** 2023-09-06

**Authors:** Jumana Muallem, Renata Voltolini Velho, Johanna Netzl, Jalid Sehouli, Sylvia Mechsner

**Affiliations:** Department of Gynecology Charité with Center of Oncological Surgery, Endometriosis Research Center Charité, Campus Virchow-Klinikum, Augustenburger Platz 1, 13353 Berlin, Germany

**Keywords:** Chronic pelvic pain, Spinal hyperalgesia, Pelvic floor muscles, Pelvic floor dysfunction, Electrostimulation

## Abstract

**Purpose:**

Chronic pelvic pain (CPP) is one of the main problems of endometriosis, leading to a significant impairment of quality of life. Understanding the pain mechanisms and the pelvic floor muscles (PFM) changes in these patients is essential to integrate additional therapeutic strategies. We hypothesize that endometriosis patients have changes in PFM and that targeted vaginal electrostimulation can be a treatment option for CPP in this disease.

**Methods:**

Fifteen patients with endometriosis and chronic acyclical pelvic pain were included. PFM electromyography with the Multiple Array Probe Leiden (MAPLe) was performed. Mapping of PFM was utilized and targeted electrostimulation of the hypertensive muscles was conducted. Control electromyography was performed afterward to evaluate the electrostimulation therapeutic effect.

**Results:**

In 12/15 (80%) patients, the myofascial trigger point could be localized by digital examination. The most frequently affected muscle was the *puborectalis* (10/15—66.7%). Most of the patients showed serious changes in the average resting tone (aRT) of PFM. aRT was significantly increased in all patients and decreased after stimulation, whereby the difference prior to and after stimulation was not significant (*p* = 0.064). The detailed separated analysis of the hypertensive muscles showed a significant (*p* = 0.026) reduction in their resting tone (hRT), after targeted stimulation.

**Conclusion:**

Vaginal electrostimulation is a promising and feasible complementary treatment option for CPP in endometriosis patients. Targeted treatment of pelvic floor dysfunction should be included in clinical trials.

## What does this study add to the clinical work

**Table:** 

Vaginal electrostimulation is a promising and feasible complementary treatment option for chronic pelvic pain in endometriosis patients. Targeted treatment of pelvic floor dysfunction should be included in clinical trials.	

## Introduction

Endometriosis is an unrecognized chronic inflammatory gynaecological disease affecting approximately 270 million women worldwide [1]. Chronic pelvic pain (CPP) is one of the main problems of this condition, leading to significant impairment of the patient’s quality of life. Patients frequently report a long diagnostic delay and a complex pain syndrome with combinations of pelvic pain and painful bladder syndrome, irritable bowel syndrome, vulvodynia, pelvic floor tenderness and dyspareunia [1, 2].

The pathogenesis of pain generation is very complex [1–3]. A combination of peripheral and central (both visceral and somatic) pain sensitization explains the high complexity of symptoms in patients with endometriosis [3]. In case of recurrent pain like dysmenorrhea, central sensitization mechanisms are activated every month, and pain-intensifying mechanisms are upregulated [4, 5]. Unfortunately, only limited data are available regarding pathological changes of the pelvic floor in endometriosis patients and a possible impact of this on pelvic pain. However, various studies on the pelvic floor muscles (PFM) with pelvic pain not caused by endometriosis showed a wide range of changes in power, ability of coordination, speed of coordination, endurance, tone and relaxation [6–9]. These data suggest that the pelvic floor may play an important role in the generation of pain in endometriosis as well [10]. Myofascial trigger points are a possible cause of pelvic pain.

Currently, the standard treatment options are not able to resolve the chronic pain situation in these women sufficiently [1, 11]. While hormonal and surgical treatment are the most common treatment options, high recurrence rates and ongoing pain after such interventions are frequent [11]. Although a Cochrane review [12] recommended nonsurgical interventions for the management of CPP in general, studies regarding the nonsurgical management of CPP in endometriosis are lacking. A thorough investigation of pelvic floor changes in endometriosis patients is necessary to better understand the pathogenesis of endometriosis-related pain and the development of additional therapeutic management strategies directly targeted at the pelvic floor. Multimodal treatment strategies should be established to improve the quality of life of women with endometriosis.

To analyze the pelvic floor function in patients with endometriosis, we performed pelvic floor muscle electromyography (EMG) with the Multiple Array Probe Leiden (MAPLe) [13]. In addition, we performed a targeted vaginal electrical stimulation of the affected hypertensive muscles as a nonsurgical pain treatment for endometriosis and CPP patients.

## Materials and methods

This prospective pilot study was conducted at the Endometriosis Centre of Charité—Universitätsmedizin Berlin, Germany, from December 2019 to May 2020.

Fifteen patients with histologically confirmed endometriosis and CPP were included (age 18–49). They suffered from CPP not responding to hormonal, analgesic, and anti-inflammatory treatment. Numerous studies have utilized this procedure on both patients and healthy individuals, and it has been shown to have low risks and side effects. Some people may experience mild tingling or twitching under the electrode area, but this sensation goes away immediately once the stimulation stops. However, individuals with severe or untreated internal, neurological, or psychiatric conditions, those who are pregnant or breastfeeding, people with cardiac pacemakers, myocardial damage, arrhythmias, and epilepsy patients were excluded from the study. We assessed the PFM of all participants before the intervention using a single-digit pelvic examination of the levator ani muscles (*Iliococcygeus* muscle, *Pubococcygeus* muscle, *Puborectalis* muscle) which were palpated for tenderness on each side. Tenderness on each side was rated by the patient as absent or present. Patients in whom a pelvic examination could not be performed (e.g., vaginismus) were excluded.

The severity of pain was documented using a standardized questionnaire with a visual analogue scale (VAS) before the intervention. The pain intensity was determined with the help of a visual numerical analogue scale (0 = no pain, 10 = strongest imaginable pain).

**Fig. 1:**
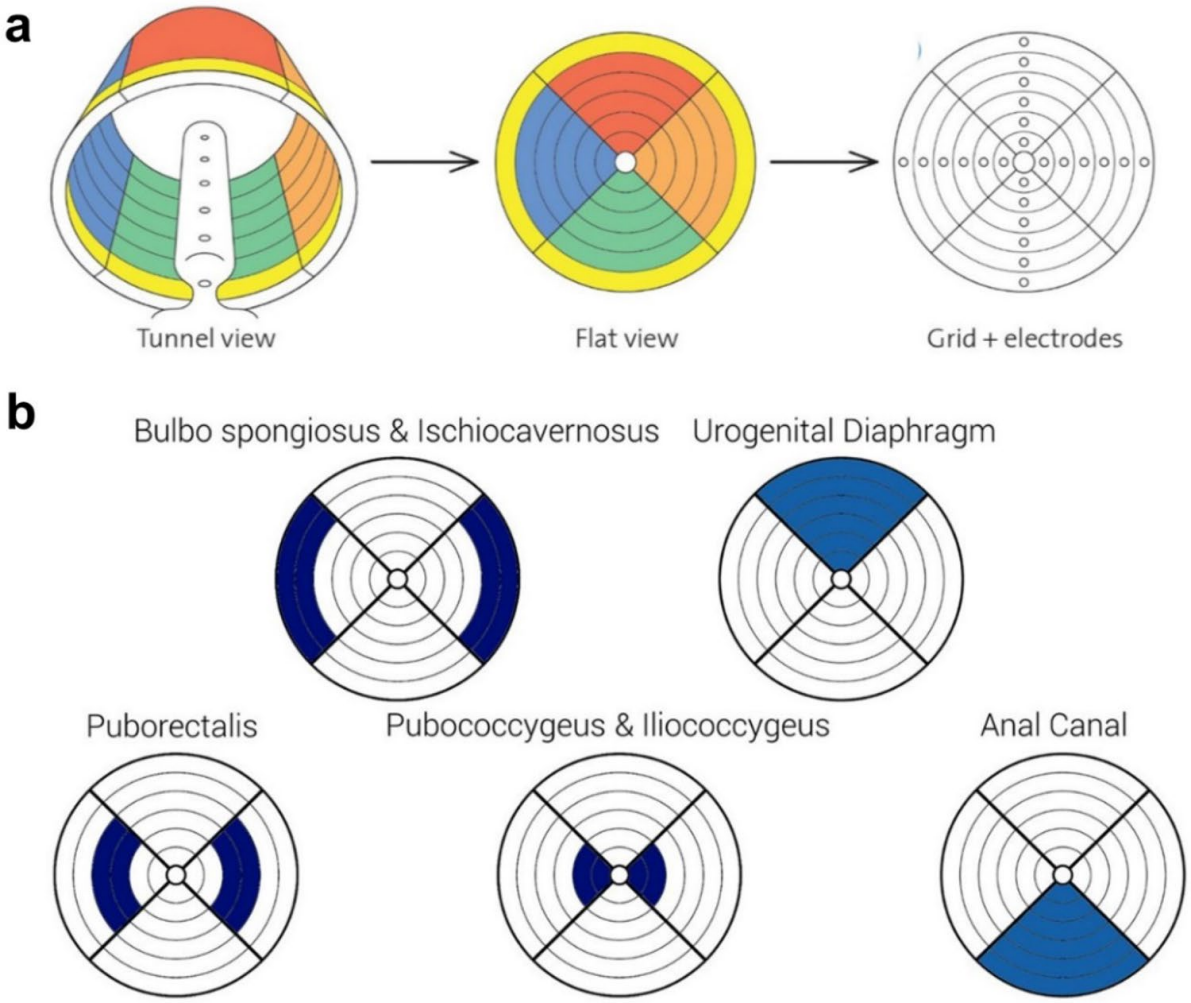
The MAPLe system. **a** The MAPLe probe has electrodes on six depths and four sides. The MAPLe grid is displayed. This is used in the figures showing the results of the analyses in this study. **b** Graphical representation the pelvic floor muscles and structures (vaginal) with respect to the MAPLe electrodes in a visualization Grid. The four compartments represent the anterior (12 o’clock), left (3 o’clock), posterior (6 o’clock) and right side (9 o’clock) of the PFM. The most outer rings are located at the most superficial parts of the PFM, the most inner ring (nearest to the center) is located at the most deeper parts of the PFM

**Fig. 2:**
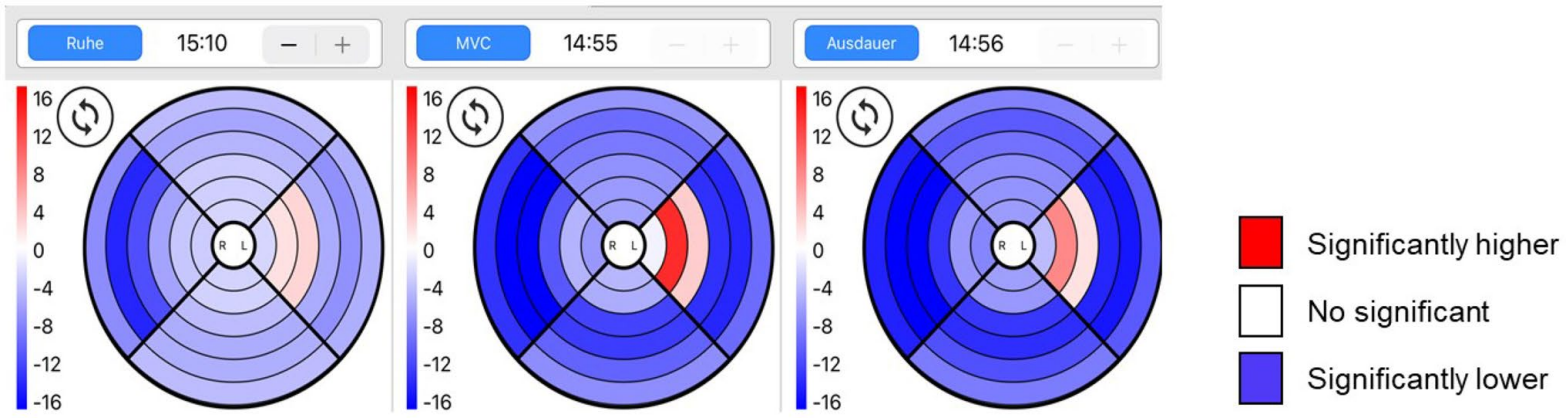
A visual representation of the PFM activity presented in a tablet application (app) developed for the MAPLe device. Color scales (white–red–blue) represent the microvolt (EMG amplitude) readings for the 24 electrodes, which are graphically presented in a “bull’s eye” pattern. Red means hypertonic (2–12 µV), blue means hypotonic (− 1 to − 8 µV), and white means normal muscle activity (0 µV)

For the EMG measurement, we utilized the MAPLe device. The probe from this device contains a matrix of 24 electrodes (six levels, 10 mm apart, on six depths and four sides) that measure EMG signals from the different layers and sides of the PFM as shown in Fig. 1. The MAPLe Probe was placed intravaginally, with a grounding electrode on the spina iliaca anterior superior. The patients were asked to perform three consecutive tasks: (i) 1 min rest, where patients were instructed to feel the pelvic floor in rest (average rest tonus—aRT); (ii) 10 maximum voluntary contractions, where patients were instructed to perform a controlled contraction and relaxation of the PFM; and (iii) 3 endurance contractions, where patients were instructed to contract the PFM at such a level that they could hold for 30 s. During these examinations, no instructions were given on how to perform a pelvic floor muscle contraction. A visual representation of the PFM activity is presented in a tablet application (app) developed for the MAPLe device (Fig. 2). Color scales (white–red–blue) represent the microvolt (EMG amplitude) readings for the 24 electrodes, which are graphically presented in a “bull’s eye” pattern. Red means hypertonic (2–12 µV), blue means hypotonic (−1 to −8 µV), and white means normal muscle activity (0 µV). In case of hypertonic/hyper-tense muscles, a RT of these muscles (hRT) was evaluated separately before and after intervention.

After the affected painful and hypertensive muscles had been identified through the process described, a targeted focal electrostimulation (intervention) based on the control gate theory [14] was applied for 10 min to these muscles as a possible treatment. To evaluate the effect of the electrostimulation on the whole PFM, a new EMG measurement at rest (aRT) of the PFM was performed.

Statistical analyses were performed using IBM SPSS Statistics, Version 26 (IBM Corp. IBM SPSS Statistics for Windows, Version 26.0. 2019). Data were described by median and percentiles. A non-parametric Spearman correlation analysis was performed to examine the association between the Resting tone (RT) before and after intervention. Two one-sample Wilcoxon sign rank tests were conducted to test if the RT before and the RT after intervention differed from the critical value for an increased RT of 2 µV [13]. Finally, a paired sample Wilcoxon sign rank test was performed to test if the RT had improved after the intervention. All these analyses were performed for the general RT (aRT) and the RT of the affected hypertensive muscle (hRT). Statistical significance was defined as *p* ≤ 0.05.

## Results

Fifteen patients were recruited from daily practice and analyzed. Demographic characteristics are summarized in Table 1. The mean age of participants was 32.93 ± 8.49 years (range 18–49 years). Three (20%) patients were parous. Most participants (12–80%) were under hormonal therapy with either a levonorgestrel-releasing intrauterine device (2), GnRHa (3), or progestogen-only pill (7). The CPP duration ranged from 5 to 15 years (7.5; P25 = 6; P75 = 10) with a median pain intensity of 6 (P25 = 5; P75 = 7).

**Table 1:** Characteristics of the study participants

Characteristics	*N* = 15
Age (years)	
Median (range)	33 (18–49)
Years of pain	
Median (range)	7.5 (5–15)
Hormone use	
Contraceptive pill	7
Intrauterine device	2
GNRH analog	3
None	3
VAS	
Median (range)	6.0 (4–8)
Pregnancy	3
Parous	3

In 12 (80%) patients, the myofascial trigger point could be localized by digital examination. In 3 (20%), it was not possible to identify the myofascial trigger point, but the tout bands of PFM. The most frequently affected muscle was the *puborectalis* (10/15—66.7%). In one endometriosis woman, the *Pubococcygeus* muscle was affected.

**Fig. 3:**
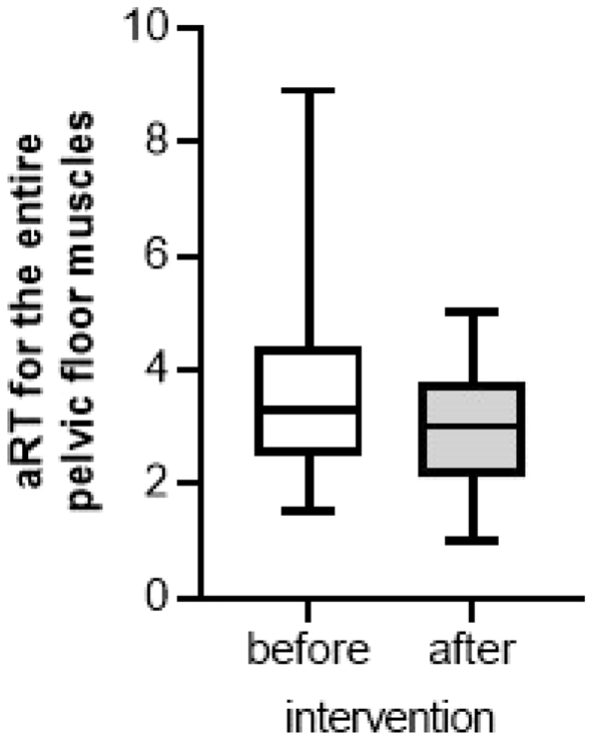
Resting tone (aRT) for the entire pelvic floor muscle before and after the intervention (*n* = 15)

Before the intervention, the median aRT was 3.30 (P25 = 2.50; P75 = 4.40), which was significantly higher than the critical value for an increased RT (*p* = 0.002). After the intervention, the median aRT decreased to 3.00 (P25 = 2.10; P75 = 3.80), but was still higher than the critical value (*p* = 0.017) (Fig. 3). All patients who initially had an increased aRT (12/15, hyper-tense muscles) still showed after the intervention (*p* = 0.05). The difference between the median aRT before and after the stimulation was not significant (*p* = 0.064).

**Fig. 4:**
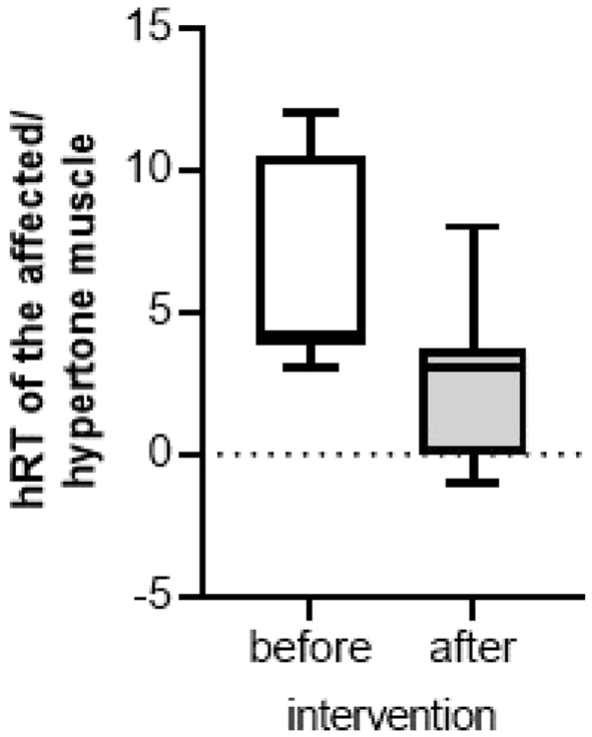
Difference in resting tone of the affected, hypertone (hRT) muscle before and after the intervention (*n* = 12)

A RT of the hyper-tense muscles (hRT—12 patients) was evaluated separately before and after the intervention. The median RT of the affected hypertensive muscle before the electrostimulation was 4.00 (P25 = 4.00; P75 = 10.50), significantly higher than the critical value for an increased RT (*p* = 0.002). After the targeted stimulation, the median hRT decreased to 3.00 (P25 = 0.00; P75 = 3.75) and did not differ from the critical value for an increased RT (*p* = 0.937). A statistical significance was obtained with the difference between hRT before and after the stimulation (*p* = 0.026) (Fig. 4).

During the targeted electrostimulation, most patients (14–93.3%) recognized the targeted area as one of the painful points. All patients (15–100%) relate the experience as pleasant; 12 (80%) felt pain relief a couple of hours later; 14 (93.3%) patients felt pain relief a day after the intervention that lasted for a couple of hours.

## Discussion

In this pilot study, a new intravaginal device was used for targeted electrical stimulation of myofascial trigger points to relieve CPP in women with endometriosis. Using MAPLe allowed us to analyze the different parts of the PFM separately. As expected, the analysis has shown a significant increase in the aRT of the PFM in this group of patients compared to the aRT in healthy volunteers in the validation study [13]. Although we did not find a significant decrease in the total aRT after the intervention, the value of *p* (0.064) is promising as it suggests that an effect of the intervention would be obtained in a larger sample or after the electrostimulation repetition, thus underlining the importance of the continuation of this study. The detailed analysis of the hypertensive muscle groups showed a higher RT (hRT) in endometriosis patients compared to healthy controls [13]. Moreover, the difference in hRT before and after the targeted stimulation was statistically significant. This showed a positive effect of the targeted stimulation in reducing the tension in these hypertonic muscles. The patients identified the localized hypertensive muscle groups according to their painful trigger points. This result is of high clinical relevance as it suggests the possibility of pain relief for a patient group heavily burdened by pain symptoms.

For the better development of treatment strategies for CPP in endometriosis, the underlying mechanisms of the pathogenesis of the disease need to be studied in more detail. One of the underestimated sources of pelvic pain is not the pelvis itself, in terms of the comprised visceral organs and the peritoneum, but the pelvic floor and its layering of different levator ani muscles. As a result of endometriosis-related pain, patients search for pain-resolving positions, however, malposition leads to asymmetric reflector contraction of the PFM, resulting in CPP and PFM dysfunction. In 2015, Stratton was one of the first who raised the possible influence of the pelvic floor on pain generation [15]. However, still, limited data on pelvic floor activity in patients with endometriosis is available.

Most of the few trials published are based on invasive therapies. Myofascial trigger points injection, with or without a topical anesthetic (wet or dry needling, respectively), is another common form of myofascial treatment being studied. Direct injection of a local anesthetic such as lidocaine is thought to diminish the ability to transmit pain signals through these hyperactive neural networks, providing additional pain relief that may be more durable than myofascial trigger points needling alone [16, 17]. Prospective studies using dry needling have not been performed in the pelvic region. Nonetheless, dry needling in other body regions has been shown to reduce pain and is non-inferior to wet needling [18, 19]. The injection of botox (a brand name for botulinum toxin) into the myofascial trigger points has also been investigated. This neurotoxin can temporarily paralyze muscles by blocking the release of acetylcholine, a neurotransmitter that signals muscles to contract. In a double-blinded randomized study, symptom improvement in 7 patients was observed. Patients from the placebo group also experienced pain relief, although the duration of the pain mitigation was longer lasting in those who received botox [20].

Seeking alternatives for a more efficient treatment of endometriosis and CPP, physical activity and exercises come to light. In a recent review [21], the authors emphasized that physiotherapy in its various forms can be an excellent complement to the gynecological treatment of endometriosis, reducing inflammation, alleviating pain, and thus significantly improving women’s quality of life. Small studies tout the effectiveness of these techniques in the treatment of myofascial pelvic pain, including one retrospective study which showed that physical therapy benefits up to 63% of patients who attempt it [22, 23]. In a recently published meta-analysis, the available evidence for the effect of physical activities and exercise on endometriosis-associated symptoms was summarized [24]. Three interventional studies involving 109 women were identified and included. Each included study found some improvement in pain intensity, stress levels, well-being, or self-image. However, due to confounding factors, the effect of physical activities and exercise alone could not be determined. Nevertheless, the authors affirm that these activities might exert a range of beneficial effects on endometriosis-associated symptoms [24].

Previous studies using electrotherapy demonstrated the benefits relieving CPP related to endometriosis. Self-applied transcutaneous electrical nerve stimulation (TENS) involves the application of adhesive electrodes to the skin with subsequent electrical stimulation of painful areas [25]. Interferential current (IFC) therapy involves the application of medium-frequency alternating currents, which are thought to increase blood flow and reduce pain [26, 27]. Seems advantageous over TENS as it generates an amplitude-modulated frequency, allowing it to penetrate more deeply than TENS. Despite the efficacy, both methodologies have been demonstrated to be similar and beneficial in endometriosis-related CPP [28].

To the best of our knowledge, only our group has evaluated a target vaginal electrical stimulation (using MAPLe). Even though our pilot study has some limitations, such as the short therapy duration (one session of electrostimulation) and the small number of participants, promising results could be seen. Vaginal electrostimulation is an encouraging and feasible complementary treatment option for CPP in endometriosis patients, and future clinical trials should include this approach. Endometriosis is an under diagnosis and undertreated complex disease. Multimodal treatment strategies should be established to improve the quality of life of these patients.
